# A Double‐Humanized Murine Model in Bladder Cancer: A Novel Preclinical Model for Cancer Immunology Research

**DOI:** 10.1002/cam4.71150

**Published:** 2025-08-13

**Authors:** Niannian Ji, Zaineb Hassouneh, Shaun Trecarten, Zhen‐Ju Shu, Jaime Furman, Tyler J. Curiel, Robert S. Svatek, Neelam Mukherjee

**Affiliations:** ^1^ Experimental Developmental Therapeutics (EDT) Program, Mays Cancer Center at UT Health MD Anderson San Antonio Texas USA; ^2^ Department of Urology UT Health San Antonio San Antonio Texas USA; ^3^ Pathology Reference Laboratory San Antonio Texas USA; ^4^ Division of Hematology/Medical Oncology at the UT Health San Antonio San Antonio Texas USA

**Keywords:** bladder cancer, double‐humanized model, patient‐derived xenograft

## Abstract

**Background:**

Bladder cancer (BC) ranks as the 10th leading cause of cancer‐related deaths in the United States. Despite significant advancements in managing BC, including antibody‐drug conjugates and immune checkpoint blockers, a significant percentage of patients fail these treatments. This underscores the pressing need to develop methods for identifying novel therapeutic targets, predicting therapy responses, and optimizing personalized treatment strategies for patients. Patient‐derived xenograft (PDX) models have the potential to address these challenges. However, these models suffer from limitations, including a consistent rate of tumor acceptance, extended time for tumor growth, and the absence of the donor's immune system.

**Methods:**

This study aimed to address these challenges through a comprehensive comparison of different PDX implantation methods, culminating in the establishment of a PDX cell line for utilization in in vivo BC preclinical models. Flow cytometry analysis and total RNA sequencing were used for comparative analysis of original tumor tissue, PDX tumor, and PDX257S cell line.

**Results:**

Tumor tissue processing techniques were optimized to enhance the rate of PDX tumor acceptance and shorten the time to tumor development. Furthermore, a PDX cell line, PDX257S, was successfully established and confirmed to exhibit more aggressive and tumorigenic characteristics compared to the original donor tumor tissue. The PDX257S cell line demonstrated faster tumor growth, higher expression of human epithelial cell adhesion molecule, increased mutation burden in BC‐associated genes, and significant alterations in BC‐related gene expression, compared to the original tumor. The PDX257S cell line was also used to establish a double humanized BC murine model that minimized graft‐versus‐host disease and is a potential platform for testing novel immune therapeutics.

**Conclusions:**

In summary, this study offers an optimized protocol for the consistent establishment of BC PDX tumors. Furthermore, it has also established a novel double‐humanized model, demonstrating the potential for drug screening and clinical prognosis in the context of BC treatment.

## 1 Introduction

Bladder cancer (BC) is a common malignancy that accounts for more than 82,000 new cases and around 16,000 deaths annually in the United States [1]. 75% of BC manifests as nonmuscle invasive BC (NMIBC); the most common treatment for NMIBC is transurethral resection together with intravesical instillation of chemotherapeutics or Bacillus Calmette‐Guérin [2, 3]. Management of BC has undergone rapid development in the last decade, and immune therapy has been a breakthrough in the landscape of BC treatment, with the FDA approval of all five checkpoint blockers targeting programmed cell death protein 1, such as pembrolizumab and nivolumab, or programmed cell death ligand 1, including atezolizumab, avelumab, and durvalumab, along with the introduction of the first antibody–drug conjugate, enfortumab vedotin, transforming the landscape of BC treatment [4, 5, 6, 7]. However, despite the success of immune therapies, complete response to immune checkpoint blockade is seen in only 20%–30% of the patients [8], highlighting the need for optimized treatment strategies and new targeted therapies. One of the current obstacles in the field is a dearth of preclinical disease models that recapitulate the biology and heterogeneity of bladder tumors.

Patient‐derived xenograft (PDX) models are generated by the transfer of tumor fragments extracted from patients into immunocompromised mice. The unique advantage of PDXs is that they are directly derived from clinical patient specimens and retain the morphology and genomic fidelity of their parental patient cancers compared with cell‐line‐derived xenograft models [9]. PDXs have been widely used in mechanistic studies and drug screening and are particularly effective in predicting tumor‐specific drug response; they can facilitate personalized treatment plans in patients [10, 11, 12, 13]. However, the PDX model has certain limitations, such as low engraftment rate, prolonged tumor development time, and the absence of a competent immune system in the host animals, rendering the platform unsuitable for immunotherapeutic studies.

To address the challenge of a lack of an immune system, humanized mice with engrafted human immune systems were developed. However, though xenografting human immune cells into immunodeficient mice is feasible, differences in donor HLA class I alleles can give rise to immune cell‐dependent spontaneous xenogeneic graft versus host disease (GVHD) [14], characterized by infiltration of immune cells, organ damage, and ultimately fatal outcomes [15]. GVHD pathologies decrease the number of viable mice in experimental studies and complicate data interpretation in disease models. One potential solution to minimizing GVHD in humanized mouse models is to simultaneously and locally (e.g., in the skin) engraft human innate immune cells and human tumors into the same mouse, thereby creating a double‐humanized mouse. Here, we refine the protocol for the consistent development of BC PDX tumors and also describe the successful creation of a double‐humanized PDX model of BC, which will allow the investigation of BC treatments in the context of a tumor‐immune system in vivo with minimal GVHD.

## 2 Materials and Methods

### 2.1 Ethics Statement

All animal experimental procedures were approved by the UT Health San Antonio Institutional Animal Care and Use Committee (IACUC; protocol #20150058AR). Any special training in animal care or handling needed in the study was provided to research staff by institutional veterinarians. Collection and use of clinical samples from Bladder Cancer Repository (BCR) patients were approved and abided by the institutional review board protocol HSC2012‐159H. Written consent forms were obtained.

### 2.2 Animal Usage and Humane Endpoint

Male and female NOD scid gamma (NSG) mice (The Jackson Laboratories, Bar Harbor, ME) were bred and housed under specific pathogen‐free conditions due to the animals' immune‐deficient background. The age of mice used for any tumor implantation ranged from 6 weeks to 12 weeks. For subcutaneous (sc) or bladder‐wall implantation, both male and female mice were used. For establishing initial PDX tumors from 59 patients/donors, each BC tumor sample was implanted into one to two NSG mice, depending on the type(s) of implantation: both orthotopic/bladder‐wall and sc implantations (one mouse each type), or only one type (for some sc implantation, one animal can have up to two tumor sites on both flanks). For any established sc PDX tumor, each continuous in vivo passage was conducted in one NSG mouse, but with no more than three passages when sufficient single cells were cryopreserved from a digested tumor. For evaluating the sc growth of PDX257S cell line after in vitro passages, one NSG mouse bearing two tumor sites on the flanks was used for any passage examined. In double‐humanized sc tumor study, mice were randomized prior to tumor challenge with normalized age distribution to minimize potential confounders (e.g., animals from the same litter/cage were challenged within a short period of time but assigned to different groups randomly by ear‐notching). Each group contained 2–4 mice (4–8 sc tumors/units) in each experiment, which was repeated 2 times independently. Power analysis was used to estimate the minimum sample size required for a single or pooled experiment. No criteria were set for including or excluding animals during data acquisition and analysis. No exclusions of any animal used and survived any procedure for experiments or data points. For double‐humanized sc tumor experiment, N.J. was aware of the group allocation while conducting the challenge/treatment and during data analysis, whereas Z.J.S. was measuring the tumors/assessing outcomes unaware of group allocation. For any histopathology evaluation of tumor tissue, the pathologist was blinded to group allocation or naïve vs. human innate immune‐cell engrafted organs or matching of any PDX tumor tissue to its original BC tumor tissue.

Humane endpoints were applied, and animals were euthanized immediately whenever any of these criteria were met: 1) any sc tumor reached over 1500 mm^3^; 2) any severe hematuria or post‐surgical infection/complications resulted from orthotopic implantation of tumor tissue/cells accompanied by worsened body condition (weight loss ≥ 20%, low body temperature, reduced mobility, etc.); 3) end of the experiment. The duration of any experiment was: 1) 8 months for initial PDX tumor establishment by orthotopic implantation of BC tumor tissue; 2) ~2 months for any actively growing sc tumor. All animals were closely monitored daily within the first 2 weeks after any surgery for bladder‐wall implantation (< 5 mm abdominal incision and closure performed under sterile conditions with anesthesia and painkiller), then every other day till the end of the experiment. No animal died due to any post‐surgical infection or complications; however, there was one death during anesthesia due to unknown sensitivity/reaction to otherwise safe anesthesia dosing (this animal was not included/counted as any data point in the result of Table 1 for tumor incidence). Mice bearing sc tumors were monitored and measured with a caliper every other day starting as early as Day 5 post‐tumor challenge or implantation.

**TABLE 1:** Comparison of various methods of tumor processing and implantation sites versus PDX tumor incidence.

Methods	PDX donor tumor size	Processing approach	Implant site	Tumor incidence	Time of visible tumor growth
I	≥ 0.2 g	Digested then mixed with MM	Bladder wall	0/11	> 200 days
II	≥ 0.2 g	Digested then mixed with MM	sc	3/27	~40 days
III	< 0.2 g	Small piece immersed in MM	sc	2/37	> 55 days
IV	< 0.2 g	Minced then mixed with MM	sc	3/15	~50 days

*Note:* Various fresh tumor tissue processing methods were used and compared either from the same patient (into more than one NSG mouse recipient) or from different patients. Tumor tissue (≥ 0.2 g) was digested into SCS and mixed with MM prior to (I) bladder wall or (II) sc injection. Small/tiny tumor tissue (< 0.2 g) was (III) chopped briefly into ~1 mm^3^ pieces and immersed directly in MM prior to sc implant, or (IV) was minced thoroughly and then mixed with MM prior to sc implantation. Tumor incidence/acceptance rate was recorded as any stable growing tumor(s) on NSG recipient(s) after each PDX from any BC patient. Time length means the average time of any accepted PDX implant that shows visible growth of tumor (~30 mm^3^).

### 2.3 BC Tumor Tissue Processing for Patient‐Derived Xenograft

As described previously [16], fresh BC tumor tissues (≥ 0.2 g) were minced and digested with 0.05% Trypsin/EDTA (Corning) at 1 g of tissue per 5 mL, plus 1 mg/mL of collagenase IV (Sigma‐Aldrich), and 0.25 mg/mL of DNase I (Sigma‐Aldrich) on a shaker at 37°C for 1 h; then processed into single‐cell suspension (SCS) in a B2‐biosafety cabinet under sterile conditions.

### 2.4 Patient‐Derived Xenograft and in Vivo Passages

SCS of processed BC tumor tissue (≥ 0.2 g) were mixed 1:1 with ice‐cold Matrigel Matrix (MM) (Corning) at 1–10 × 10^6^ cells and injected into the bladder wall (50 μL) through a small and sterile incision (< 5 mm) under anesthesia using a mixture of Ketamine/Xylazine/Acepromazine (80, 8, and 1 mg/kg, respectively; JHP pharmaceuticals/LLOYD Laboratories, Akorn/Boehringer Ingelheim) or subcutaneously on the flanks of male and/or female NSG mice (hair on the lower back were carefully shaved prior to injection). When fresh tumor tissue is small/tiny (< 0.2 g), it was briefly chopped/minced first; then a small piece (≤ 1 mm^3^) was either directly immersed in MM prior to sc implantation or was further minced and then mixed with MM prior to sc injection/implantation into the flanks of NSG mice. For certain tumor tissue with a sufficient amount (≥ 1 g), more than one processing method with both implantation routes (orthotopic/bladder‐wall or sc) was applied for comparison as well as for maximizing resources. Mice challenged with human BC tumor orthotopically were monitored for body condition and checked for hematuria by urine strip (TECO Diagnostics) for up to 8 months before the mice were sacrificed and the bladders were collected for H&E pathological examination of any established PDX tumor infiltration/growth. PDX sc tumor growth was followed and measured by a caliper weekly, then 2–3 times a week once tumors were visible (≥ 30 mm^3^; length × width × width/2). Any sc PDX tumors reaching over 200 mm^3^ were harvested and digested into SCS as described previously for BC tumor tissue, then implanted/injected subcutaneously into the flank of new NSG mouse recipients for in vivo passages. A similar process was repeated for continuous in vivo passages of sc PDX tumors. Extra SCS from PDX tumors were analyzed for human epithelial cell adhesion molecule (EpCAM) expression by flow cytometry, and the remaining SCS were cryopreserved in our BC repository bank. For H&E pathological examination, patient tumor tissue specimens and PDX tumor tissue samples were immersed in 10% buffered formalin for 24 h, then in 70% EtOH, and sent to the clinic/hospital or institution‐associated pathology lab for processing into paraffin‐embedded blocks and finally H&E slides prior to pathological evaluation. Some tissue sections were also stained and examined for human Ki67 expression using immunohistochemistry staining with an anti‐Ki67 antibody (clone 30–9; Roche). Images of stained tissue sections were taken by a Nikon microscope with a camera, captured, and processed by Zens software.

### 2.5 PDX Cell Line Culture and Characterization

Adherent PDX257S cell line was cultured from PDX tumor SCS and maintained in complete DMEM media (Thermo Fisher Scientific) containing 10% fetal bovine serum (FBS, HyClone/Thermo Fisher Scientific), penicillin/streptomycin (Corning), and fresh L‐Glutamine (Corning) at 37°C, in 5% CO_2_ and for at least 5 passages before being used in the mouse model. PDX257S cell line was also analyzed for human EpCAM expression by flow cytometry and sent to the American Type Culture Collection (ATCC) for authentication and confirmation of human origin by STR profiling.

### 2.6 Mouse Tumor Models Using PDX257S Cell Line

PDX257S cells were digested, harvested, and injected subcutaneously at 1 × 10^6^ cells in 100 μL phosphate‐buffered saline (PBS) at both flanks of NSG mice. Tumor volume was measured 3 times per week by a caliper and calculated using the formula: volume = length × width × width/2 (mm^3^). For the double‐humanized sc tumor model, cryopreserved autologous human peripheral blood mononuclear cells (PBMCs) of PDX257S donor were thawed using 1× Anti‐Aggregate Wash (C.T.L.) to ensure maximal viability and recovery. Then human innate immune cells (mixture of γδT cells and NK cells) were expanded from the above PBMCs in vitro for 2 weeks as previously described [17, 18]. On Day 14 of expansion, 2 × 10^6^ innate effector cells were mixed with 1 × 10^6^ of PDX257S cells at an effector: target ratio of 2: 1 in 100 μL of PBS and injected subcutaneously per flank and both flanks of NSG recipients as previously described [16], while the control group only received 1 × 10^6^ of PDX257S cells. Recipients of innate effector cells were monitored for signs of GVHD by weekly measurement of body weight and pathological examination of the liver and spleen up to Day 40 postengraftment.

### 2.7 Immune Analysis by Flow Cytometry

A representative portion of digested and processed tumor tissue SCS from sc PDX passages or sc challenge with PDX257S cell line was stained with fixable viability dye (FVD‐eFluor455UV, Invitrogen) and human EpCAM flow cytometry antibody (clone 9C4, BioLegend) as described previously [16]. Donor PBMCs, in vitro expanded innate effector cells, and peripheral blood, spleen, and liver from human innate effector NSG recipients were stained with FVD and fluorochrome‐conjugated monoclonal antibodies (mAbs): anti‐human CD45 mAb (clone HI30, BioLegend), anti‐human CD3 mAb (clone HIT3a, BioLegend), anti‐human γδT mAb (clone 11F2, BD), and anti‐human CD56 mAb (clone 5.1H11, BioLegend) as previously described [17]. For the in vitro cytotoxicity assay, PDX257S cells were stained with CFSE prior to co‐culture with Day‐14 expanded innate effector cells for 4 h at 37°C, followed by flow cytometry staining of FVD as previously described [17]. Fluorescence minus one control was included for each marker, with a gating threshold of ≤ 1% for the positive population. Samples were analyzed on the LSRII cytometer (BD) using FACS Diva Software v. 8.0.1 (BD) and FlowJo Software (v.10).

### 2.8 Total RNA‐seq of BC Cancer Tumor Samples and Xenograft Primary Tumor Tissue, and Cell Line

Formalin‐fixed paraffin‐embedded patient (PDX donor) tumor tissue block from the clinical pathology lab was sectioned and sent to Novogene Inc. (Sacramento, CA) for total RNA extraction and subsequent processing. Total RNA was also extracted from PDX tumor SCS and PDX257S cell line using RNeasy Mini Kit (QIAGEN) and sent to Novogene for further processing. All RNA QC, library construction, RNA sequencing (minimum 6G of data), and bioinformatic analysis were done at Novogene Inc.

## 3 Statistical Analysis

Tumor growth curves between groups were compared with a two‐way analysis of variance (ANOVA). For RNA‐seq data, to compare any two groups with only one variable, the D'Agostino and Pearson omnibus normality test was used; if *p* > 0.05, then a parametric t‐test was used; if *p* < 0.05, then a nonparametric Mann–Whitney test was used. If one group of data passed normality and the other did not, a nonparametric test was used; if any group had *n* < 7 (*N* too small), unless there were substantial outliers in the data sets (nonparametric distribution), it was considered to have passed the normality test. *p* values are two‐sided, and *p* < 0.05 was considered statistically significant. Statistical analyses were performed with GraphPad Prism versions 5 and 6.

## 4 Results

### 4.1 Variations in Tumor Processing and Implantation Methods Affect the Acceptance Rate of PDX Tumors

**FIGURE 1:**
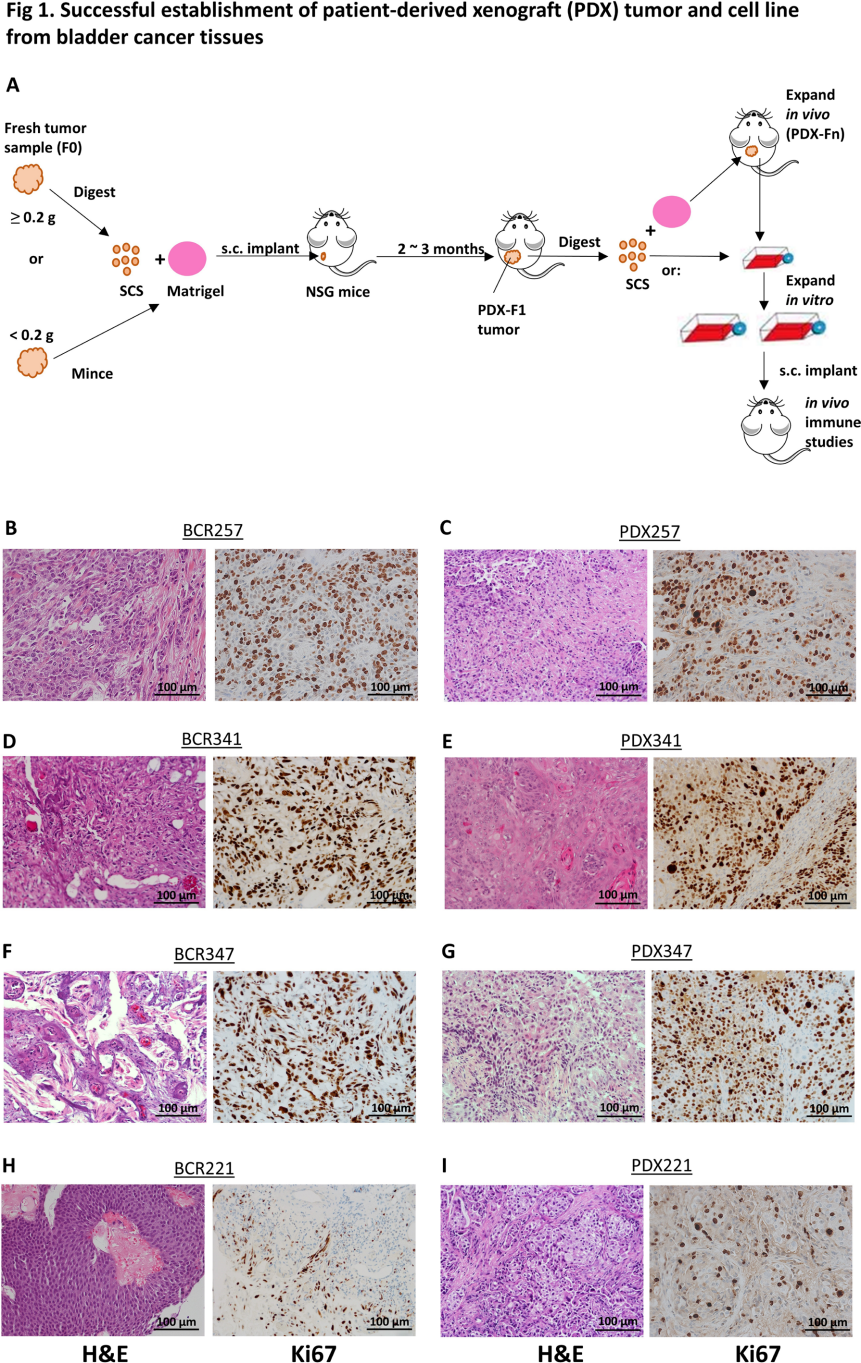
Establishment of patient‐derived xenograft (PDX) tumors and one cell line from BC tissues. (A) Brief scheme of establishing PDX tumor and subsequent cell line from BC tumor tissue. Tumor SCS from each BC patient was processed as described in Materials and Methods and implanted subcutaneously with MM into NSG mice. Original BC tumor tissue from the patient is defined as F0, and the first in vivo tumor implant grown on NSG recipient mice is defined as a PDX‐F1 tumor. PDX tumors grown from continuous in vivo implants/passages are defined as Fn. SCS, single‐cell suspension. (B–I) Examples of comparisons of original donor bladder tumors from representative patients to established PDX sc tumors, with pathological confirmation. Shown are examples approximately after 1.5 months of PDX implantation. Pathological H&E staining (left panels) and human Ki67 staining (right panels) were performed on the original patient tissue specimens (B, D, F, and H) and corresponding/matching PDX sc tumors from NSG recipients (C, E, G, and I). Shown are examples of invasive high‐grade tumors (B), invasive tumors with moderate squamous differentiation (D), high‐grade invasive tumors with squamous differentiation (F), and noninvasive low‐grade papillary tumors (H) at 20× magnification (scale bar = 100 μm).

To compare and evaluate the impact of various tumor‐processing approaches versus two different implantation sites (sc vs. orthotopic) on PDX tumor acceptance rate, fresh tumor tissue specimens from 59 patients were processed and implanted into male and/or female NSG mice. Tumor acceptance was not observed via orthotopic implantation in the first 11 tumors; therefore, subsequent test/implantation with this method was ceased (Table 1, Method I). For sc tumor implantation, using tissues that were either digested into SCS or minced thoroughly, yielded a higher PDX tumor acceptance rate (~11% or ~20%; Table 1, Methods II and IV) compared with the direct implantation of a small piece of tissue (~5%; Table 1, method III). Typically, it took up to 2 months for any accepted PDX implants to exhibit visible tumor growth (~30 mm^3^) in this study (Table 1; Figure 1A).

### 4.2 Establishment of the Tumor‐Derived PDX257S Cell Line With Aggressive and Tumorigenic Features

PDX tumors were subcutaneously implanted in NSG mice, and consecutive in vivo passages were carried out in parallel to establish PDX cell lines in vitro from each primary PDX tumor (Figure 1A). Pathological examinations were also performed on established PDX tumors and compared with the original BC tumors from PDX donors (Figure 1B–I: left panels, H&E staining; right panels, human Ki67 staining; Table S1). These donors included patients with either invasive high‐grade papillary urothelial carcinoma (Figure 1B), invasive tumors with keratinizing and moderate squamous differentiation (Figure 1D), invasive high‐grade tumors with squamous differentiation (Figure 1F), or noninvasive papillary tumors (Figure 1H and Table S1). Our analysis revealed consistent and comparable pathological features between the original patient bladder tumors and the corresponding PDX tumors harvested from NSG recipients (Figure 1B–I).

**FIGURE 2:**
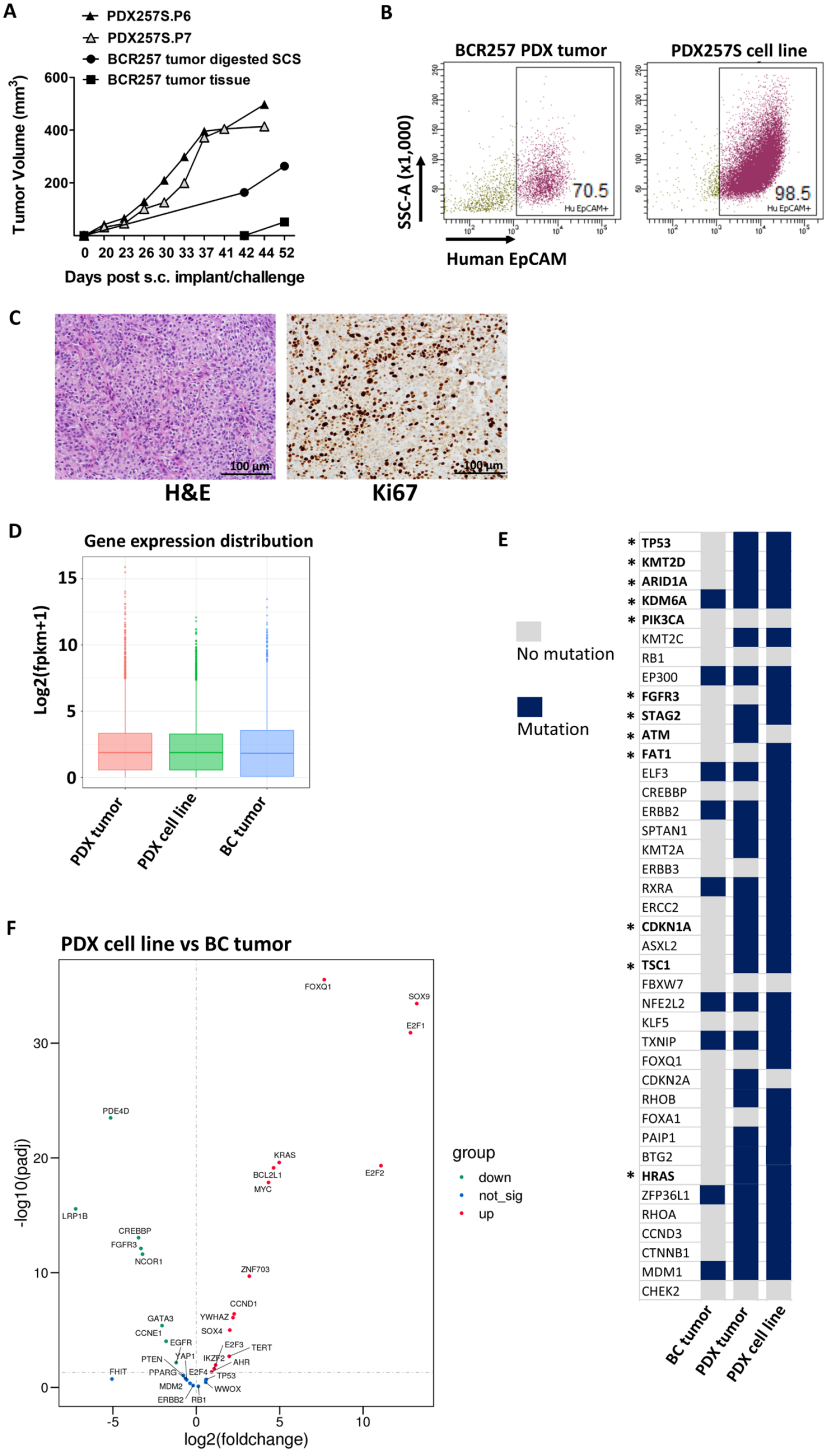
Establishing a PDX tumor cell line that is more aggressive in vivo compared with sc PDX tumor implant, and exhibits more BC tumorigenic features in mutation burden and transcriptome. (A) Growth curve of PDX‐F1 sc tumors implanted in NSG mice from patient F0 tumor tissue processed with two different methods, as shown in Table 1, Methods II vs. III, or sc tumors in NSG mice challenged with PDX257S cell line of different passages (P6, filled triangles; P7, gray triangles). (B) Flow cytometry analysis of PDX tumors from F1 implant and from established tumor cell line PDX257S. Shown are representative dot plots gated from live singlets of either digested tumor SCS or cell line SCS. (C) Pathological examination of sc tumor derived from the PDX257S challenge. Shown are examples of H&E (left panel) and human Ki67 staining (right panel) at 20× magnification (scale bar = 100 μm). (D–F) Total RNA was extracted for RNA‐seq for transcriptome profiling and gene expression analysis. (D) To compare gene expression levels under different conditions, the distribution of gene expression levels and fragments per kilobase of transcript per million mapped reads (fpkm) among different samples is displayed by boxplots. (E) Increased BC‐associated gene mutation load in PDX tumors and cell lines was found in comparison to donor tumor tissue. Gene names are in bold, and asterisks represent oncogenic drivers or putative/potential drivers for BC. (F) Comparison of gene expression level (fpkm) between PDX cell line and original BC tumor tissue. Shown is a volcano plot indicating changes in the expression of those genes with relevance found in BC from published TCGA studies (either gene copy number or expressional changes).

A stable adherent PDX tumor cell line—named PDX257S—was successfully established from the early passage of the PDX tumor of one donor (BCR257) through in vitro culture and expansion (Figure 1A). Both tumor‐digested SCS and tumor tissue from the same donor demonstrated tumor growth within 2 months of sc implantation (Figure 2A, filled circles and squares; Method II versus III of Table 1), although the SCS gave rise to a faster‐growing tumor than the undigested tumor tissue (Figure 2A, circle vs. square). Importantly, subsequent sc tumors derived from the PDX257S cell line grew at a higher rate than the initial PDX tumor (~400 mm^3^ vs. ~200 mm^3^ by Day 42; Figure 2A, triangles vs. circle). High expression of the human tumor‐initiating cell/cancer stem cell marker EpCAM [19, 20] was detected in PDX257S compared to the PDX tumor (> 98% vs. ~70%: Figure 2B). Pathological examination revealed PDX257S‐derived sc tumors with some squamous differentiation and highly proliferative features (Figure 2C). The human origin of the PDX257S was also validated via short tandem repeat (STR) profiling, though it was not found to be related to any established human cancer cell lines in the database (matching score < 85%; Figure S1). PDX tumors derived from other patient donors that failed to establish stable PDX cell lines in vitro consistently exhibited lower expression of human EpCAM compared to PDX257 tumor (below 30% vs. 70%; Figure S2 vs. Figure 2B), even after several successive in vivo passages.

Total RNA sequencing was performed on the bladder tumor tissue from patient donor BCR257, the corresponding PDX tumor, and the derived PDX257S cell line for a comparative analysis of their transcriptomes. No significant differences were found in the overall distribution of gene expression levels among the three RNA samples (Figure 2D, medium lines). We also analyzed the mutational burden in each of these three samples using a list of genes previously reported to have a significantly higher incidence of mutations in BC tumor tissue based on TCGA studies [21, 22, 23]. Small nucleotide polymorphisms (SNPs) were detected in eight genes, and an insertion/deletion was identified in the muscle‐invasive bladder cancer (MIBC) progression‐regulator ZFP36L1 gene [24] in the original BC tumor tissue of the PDX donor. These genetic variations were observed to persist in the PDX tumor and the derived PDX257S cell line (Figures 2E and S3). However, in the PDX tumor and PDX257S cell line, new SNP and/or insertion/deletion mutations were detected in 25 other genes (Figure S3), including TP53, KMT2D, ARID1A, and KDM6A genes, which were previously found with high mutational incidence (≥ 25%) among urothelial bladder carcinoma and/or MIBC tissue samples [21, 22]. Furthermore, mutations in bladder cancer oncogenic drivers such as TP53, HRAS, and CDKN1A were present in both the PDX tumor and PDX257S cell line but not in the original bladder cancer tumor. In contrast, mutations in the strong oncogenic driver FGFR3, in the form of SNPs and insertion/deletions, were exclusively found in the PDX257S cell line (Figures 2E and S3). Additionally, we also analyzed the expression of 33 genes that exhibited significant changes in gene expression, including amplification, deletion, upregulation, or downregulation in bladder tumor tissue from previous studies [21, 22, 23, 25, 26, 27, 28]. Out of the 33 genes analyzed, 9 exhibited similar expression levels between the PDX cell line and the donor tumor tissue; these genes include TP53, PTEN, RB1, MDM2, ERBB2, and E2F4 (Figure 2F). The expression of 15 other genes was significantly higher in the PDX cell line compared to the donor tumor tissue. Among them, 13 genes, including AHR, BCL2L1, CCND1, KRAS, SOX4, MYC, and E2F family genes, were previously reported to have increased expression in BC tissue [21, 22, 29, 30], and their alterations, either through amplification or upregulation, are linked with bladder tumor initiation or progression. Interestingly, the cancer stem cell marker SOX9 gene [26, 31, 32] expression level was also increased in the PDX cell line compared with the donor BC tumor. On the other hand, the expression of 8 other genes was significantly decreased in the PDX cell line compared to the donor BC tumor. Among these genes, PDE4D, LRP1B, CREBBP, and NCOR1 were previously associated with deletions in BC tumor tissue [21, 22], which favors tumorigenesis or progression (Figure 2F). Consistent with the PDX cell line, the PDX tumor not only exhibited similar expression levels for TP53, PTEN, and RB1 genes when compared with the PDX donor tumor tissue but also increased expression of genes such as E2F family, BCL2L1, KRAS, MYC, CCND1, and TERT genes as well as decreased expression of genes such as PDE4D, LRP1B, NCOR1, and CREBBP genes, which favors the tumorigenic process (Figure S4). Moreover, the BC subtype transcriptome analysis [22] indicates that the original donor BC tumor tissue expresses only a few genes related to the luminal/smooth muscle subtype (GATA3/DES), while the PDX cell line differentiated into a basal subtype (KRT5/6A and COL17A1). Notably, neither the original donor BC tumor nor the PDX cell line exhibited features of the neuronal‐differentiation subtype or the EMT/Claudin subtype (Figure S5).

Gene ontology (GO) enrichment analysis (Figure S6) revealed that the most significant differential gene expression between the PDX tumor and the original donor BC tumor is associated with cellular components or molecular functions such as plasma membrane protein complexes, extracellular matrix, cell–cell junctions, and mitochondrial inner membrane (Figure S6A–C). In contrast, the most differential expression between the genes of the PDX cell line and the donor BC tumor is associated with mitochondrial protein complexes and membranes (Figure S6D–F), along with some changes in signaling pathways such as protein kinase activity/regulation (Figure S6F).

### 4.3 Successful Establishment of a Double‐Humanized sc PDX Tumor Model With Autologous Innate Immune Cells

**FIGURE 3:**
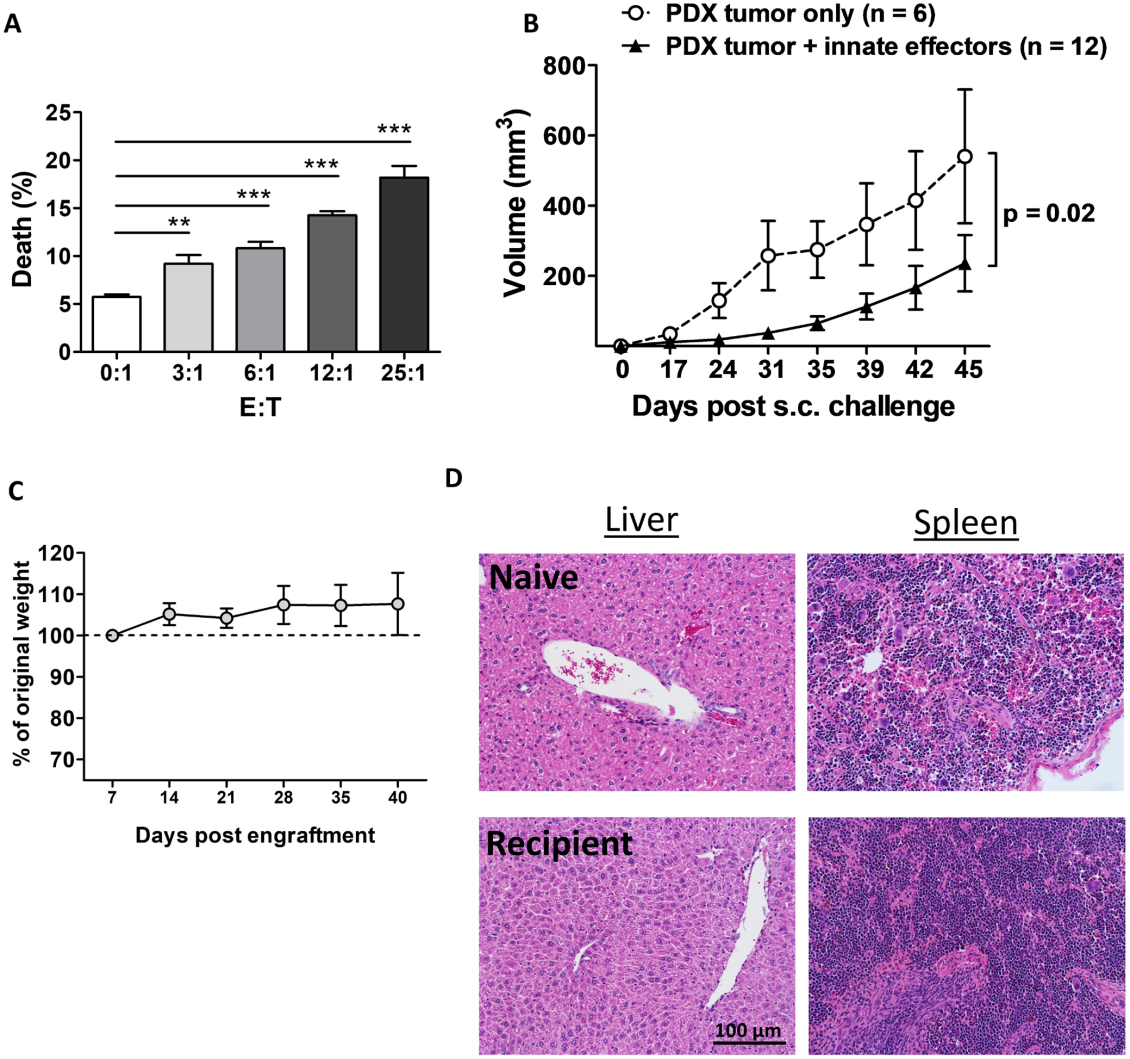
Innate immune effector cells can suppress PDX tumor growth in a double‐humanized model. Innate effector cells cultured from thawed BCR257 donor PBMCs were mixed with PDX257S cells for (A) in vitro cytotoxicity at various effector: target ratios, or (B) injected subcutaneously together with PDX257S cells (ratio 2:1) into NSG mice for in vivo cytotoxicity assay. (C) body weights of mice were monitored and recorded weekly till day 40 post‐human innate effector cell engraftment, when (D) H&E staining was performed on the liver and spleen. (A) representative results of two independent assays (*t*‐test; **p < 0.01; ***p < 0.001). (B) pooled data from two independent experiments (*n* = number of PDX257S tumors; PDX tumor only/control group, *n* = 6; PDX tumor + innate effectors, *n* = 12, mean ± SEM). ANOVA was run to compare two tumor growth curves and found significance (*p* = 0.02). (C–D) representative results from two independent experiments (*n* = 3–5 mice per experiment).

Given the success and reproducibility of the sc tumor model in NSG mice challenged with the stable and immortal PDX257S cell line (Figure 2A), an expanded double‐humanized model was explored. This novel model included the adoptive transfer of autologous innate immune effector cells (γδT cells and NK cells), which were expanded from autologous PBMCs of the PDX257S donor (Figure S7A,B). Innate effector cells had significant cytotoxicity against PDX257S tumor cells in vitro (Figure 3A). Furthermore, tumor growth in NSG mice challenged with PDX257S cells was significantly inhibited in the group that received sc coinjection of autologous innate immune cells, compared to the control group (Figure 3B). Significantly, NSG mice receiving human innate immune cells did not exhibit substantial GVHD. This was indicated by stable body weight over time (Figure 3C) and minimal infiltration of human innate effector cells in the liver (less than 0.5% of total live cells), as observed in both H&E staining and flow cytometry analysis by day 40 post‐engraftment (Figure 3D, left panels, and Figure S7C). However, there was a slight increase in infiltrates observed in the spleen (less than 3% of total live cells), as shown in Figures 3D and S7C. While tumor growth was not entirely suppressed in NSG mice that received autologous immune cells, this humanized model still proves valuable for testing and evaluating treatments or drugs aimed at enhancing or reinvigorating innate immune effector functions to further restrain tumor growth.

## 5 Discussion

The development and application of PDX models have proven to be a valuable platform for translational cancer research over the past decade [9]. While approximately 80 PDX models for BC have been reported [11, 33], many encounter challenges such as low tumor acceptance rates and extended timeframes for tumor development, averaging around 10 months. Additionally, variations in tumor grade, stage, phenotype, and immune profile contribute to significant variability in retaining the original characteristics of clinical specimens, which are essential for adequately reflecting the heterogeneity of bladder tumors [33, 34, 35]. Although PDX implantation of tumor tissue in the renal capsule may increase the acceptance rate in immune‐incompetent mice [36, 37], it is an invasive process that can also lead to certain complications in the animal, such as undesirable metastatic spread into the liver and peritoneum [38] or lymphomagenesis [37, 39]. The sc implantation, on the other hand, has been the most widely used PDX site for BC tissues; however, tumor acceptance/success rates have been inconsistent and are inconsistent among different studies [40, 41, 42, 43, 44, 45]. Importantly, no systematic study has addressed or compared the outcomes of different processing approaches of tumor tissue, for example, direct implantation of small tissue versus digested tumor SCS.

Our study first focused on evaluating the PDX tumor acceptance rates of fresh BC tumor tissue of varying sizes. This was especially critical for small‐sized specimens that could not be processed by routine digestion into SCS. We also explored different tissue processing methods and instillation routes, including sc and bladder wall/orthotopic implantation. Our findings indicate that all sc implant methods exhibit better tumor acceptance rates compared to orthotopic implantation. Although sc challenge may not fully replicate the tumor microenvironment (TME) compared to sub‐renal/renal capsule and orthotopic implantation, it remains the most widely used method due to its ease of challenge and tumor measurement [11, 37, 43, 46]. While the majority of studies of sc PDX conducted the graft by directly implanting a small piece of BC tumor tissue [41, 42, 43, 44, 45], our study demonstrates that implanting either a fully minced small piece of tissue or a representative portion of a homogeneous single‐cell suspension derived from a larger and digested tumor tissue yields higher tumor acceptance rates better than direct implantation of undigested piece(s) of the tumor specimen tissue. Although the overall tumor acceptance rates in this study are either similar to or not higher compared to some other reported studies of sc PDX for BC [42, 45], the established PDX tumors took a shorter time to grow (~2 months vs. generally up to 10 months in other studies). Although MIBC tumor tissue may have yielded a higher PDX tumor uptake rate than NMIBC tissue [42, 45], we did not differentiate or compare the stages (NMIBC or MIBC) of tumors used for PDX in this study due to less than 10 cases of successful PDX implantation.

In recent years, ex vivo three‐dimensional organoids have emerged as a promising alternative for in vitro cancer‐targeted drug testing, offering a physiologically relevant system compared to standard cancer cell lines. This approach is particularly advantageous in capturing the complexity of patient tumors. However, despite these advancements, the successful implantation of PDX tumors can still enhance and accelerate the subsequent growth and expansion of organoids, surpassing the speed achieved when establishing them directly from primary tumor tissue.

This study also successfully generated a stable tumor cell line, PDX257S, derived from one of the successfully established PDX tumors. PDX257S was confirmed to be of human origin; however, interestingly, it displayed heightened aggressiveness and tumorigenic features and accelerated in vivo tumor growth in NSG mice when compared to the initial PDX implantation of the original tumor. To investigate this increased tumorigenic potential of PDX257S, a comprehensive phenotypic and transcriptome analysis was carried out. Compared to the original tumor, PDX257S exhibited a significant three‐fold increase in mutational burden, involving more oncogenic drivers, along with elevated expression of EpCAM, a recognized marker for cancer stemness associated with a more aggressive subtype of BC tumor [19, 20]. Importantly, some mutations did not carry over from the PDX tumor to the PDX257S cell line, possibly indicating that the cell line originated from a subset of one or more clones within the original tumor. On the other hand, it also displayed alterations in the expression of key genes, including SOX9, E2F family, KRAS, BCL2L1, and MYC, indicative of a predisposition for BC tumor initiation, stemness, and progression [21, 22, 25, 26]. Lastly, PDX257S showed reduced GATA3 expression and increased KRT5/6A expression, suggesting a potential shift from luminal to basal subtype resulting in increased aggressiveness [47]. The subtype shift and higher mutational burden observed in the PDX257S line may contribute to its increased susceptibility to immunotherapy compared to the original tumor. However, this cannot be tested or verified in vivo due to feasibility constraints and the unavailability of the original tumor in viable tissue form. Additionally, differential expression of certain genes—such as those associated with mitochondrial membranes, protein complexes, or enzymatic activity—in the PDX257S line relative to the original tumor may reflect stress responses induced by cell culture and adaptation to the in vitro environment. Despite the differences with the original tumor, this patient‐derived cell line presents a valuable model for the study of aggressive BC tumors, offering insights into potential therapeutic targets and understanding of bladder tumor progression.

Subsequently, our investigation delved into the potential utilization of PDX257 for the development of a humanized BC model. Initial attempts reported by other studies to create humanized mice involved the direct transfusion of human PBMCs into immunodeficient mice to mimic the anti‐tumor immune response when immune‐modulating/T cell‐enhancing antibodies were administered [48, 49]. Unfortunately, these studies were discontinued within a few weeks due to the development of robust GVHD. Additionally, the challenge of obtaining human leukocyte antigen (HLA)‐matched PBMCs and PDXs, unless both are sourced from the same patients, presented a significant obstacle. Another study investigated an alternative approach—based on a method employed previously in a melanoma PDX model—to circumvent or slow down GVHD [50]. This involved initially implanting patient tumor fragments into mice with competent immune systems, allowing for initial immune editing. Subsequently, the PDX was expanded into a sufficient number of humanized mice. The authors of this study noted a slower growth of the humanized tumor compared to its growth following implantation in NSG mice [50], a trend consistent with our findings. To address this challenge, we established a double‐humanized PDX model through the sc challenge of PDX257S cells and localized administration of human innate immune effector cells (γδT cells and NK cells) directly into the PDX tumor. This approach minimized the systemic GVHD observed in immunocompromised NSG mice and extended long‐term survival rates in these mice. Therefore, these humanized models will serve as valuable tools for testing drugs or novel treatments that augment effector immune cells, potentially suppressing tumor growth [16].

Our study has the following limitations: 1) depending on the individual tumor implanted, this model can be both time and labor intensive, especially compared to the standard challenge of established cell lines; 2) transcriptome profiling of the PDX257S cell line revealed not only an increase in mutational burden but also a phenotypic shift from a luminal to a basal subtype compared with the original donor tumor specimen, which could limit the interpretation of results in correlation to its original in vivo tumor, although co‐administration of innate immune cells may recapitulate a more relevant TME; 3) the majority of the TME of this PDX model is still murine. Therefore, the development of a double‐humanized metastatic model by transferring human endothelial cells and autologous immune cells can be the next step for translational immunotherapy studies.

## 6 Conclusions

In conclusion, our sc double‐humanized PDX model offers a reliable and reproducible screening platform for standard and personalized treatment strategies for patients suffering from BC within a reasonable timeframe without compromising its clinical relevance. This study also has optimized methods for tissue processing and sc implantation, tailored for tumor specimens of varying sizes, which further increases its clinical utility. We expect that this innovative PDX model will prove valuable for investigating the mechanisms underlying the progression of BC and for choosing treatment strategies for aggressive cases of BC.

## Author Contributions

**Niannian Ji:** conceptualization, methodology, data curation, investigation, validation, formal analysis, supervision, funding acquisition, writing – original draft, writing – review and editing. **Zaineb Hassouneh:** data curation, formal analysis, visualization, writing – review and editing. **Shaun Trecarten:** data curation, validation, writing – review and editing. **Zhen‐Ju Shu:** methodology, data curation. **Jaime Furman:** data curation, formal analysis, visualization, writing – review and editing. **Tyler J. Curiel:** methodology, resources, funding acquisition. **Robert S. Svatek:** conceptualization, methodology, supervision, funding acquisition, writing – review and editing, writing – original draft. **Neelam Mukherjee:** conceptualization, investigation, supervision, funding acquisition, writing – original draft, writing – review and editing, methodology.

## Consent

All authors have consent for publishing the data from these studies. The protocol used in this study has not been registered.

## Conflicts of Interest

The authors declare no conflicts of interest.

## Supporting information


**Figure S1:** Confirmation of PDX257S cell line with human origin by STR profiling. PDX257S cell line was submitted, and STR profiling was done at ATCC with confirmation report of human origin. STR profiles were also searched in the Cellosaurus database for potential matches to any human cell line (> 85%), as shown above.

**Figure S2:** PDX tumors that did not establish cell lines had low expression of human EpCAM. (A) Donor BCR347 and (B) donor BCR341 PDX early passage tumors (F1 or F2) had relatively low (< 50%) human EpCAM expression by flow cytometry analysis, whereas no subsequent cell lines were generated.

**Figure S3:** Increased BC‐associated gene mutation load was found in the PDX tumor and cell line compared with the original tumor from the donor. Gene mutation analysis was performed for small nucleotide polymorphism (SNP; left panel, red blocks) and insertion/deletion type of mutations (right panel, purple blocks) for PDX tumor, cell line, as well as original donor tumor tissue. Gene names marked with asterisk represent oncogenic drivers or putative/potential drivers for BC.

**Figure S4:** Comparison of gene expression level (fpkm) between PDX tumor and original BC tumor tissue. Shown is a volcano plot indicating changes in the expression of those genes with relevance found in BC from published TCGA studies (either gene copy number or expressional changes).

**Figure S5:** Transcriptome profiling and comparison of PDX tumor, cell line versus original BC tumor tissue in BC subtypes. Shown is an expressional heatmap plotted on fpkm value, with clusters of genes indicating different subtypes of BC.

**Figure S6:** Differential gene expression gene ontology (GO) enriched analysis of PDX tumor, cell line versus original BC tumor. Shown are GO enrichment analysis scatter plots for (A) PDX tumor vs. BC tumor and (D) PDX257S cell line vs. BC tumor. The abscissa in the graph is the ratio of the differential gene number to the total number of differential genes on the GO Term, and the ordinate is GO Term. padj: adjusted *p*‐value. Directed acyclic graphs (DAG) are also shown for (B‐C) PDX tumor vs. BC tumor or (E‐F) PDX cell line vs. BC tumor using GO terms under (B & E) cellular component and (C & F) molecular function. Each node represents a GO term, and the box represents the enrichment level of TOP5 GO Terms. The depth of the color represents the degree of enrichment; the darker the color is, the higher the enrichment degree is. Each node shows the name of the term and the padj of enrichment analysis.

**Figure S7:** Transfer of human PBMC‐expanded innate effector cells did not cause substantial GVHD. (A) Examples of flow cytometry gating strategy with (B) absolute number (AN) of innate effector cells (γδT cells and NK cells) per 106 PBMCs before and after expansion in vitro for 14 days. (C) Innate effector cells were injected subcutaneously together with PDX257S cells (ratio 2:1) into NSG mice for an in vivo cytotoxicity assay. By Day 40 post‐human innate effector cell engraftment, peripheral blood (PB), spleen, and liver were collected for flow cytometry analysis to detect human donor cells besides pathology staining.

**Table S1:** Donor tumors with successful PDX implants were mostly from invasive and/or high‐grade urothelial carcinoma.

## Data Availability

The data that supports the findings of this study are available in the Supporting Information of this article.
