# Supplementary figures and images for: A Double‐Humanized Murine Model in Bladder Cancer: A Novel Preclinical Model for Cancer Immunology Research
Source: Cancer Med. 2025 Aug 13;14(15):e71150. doi: 10.1002/cam4.71150 (PMC12344512; doi:10.1002/cam4.71150)

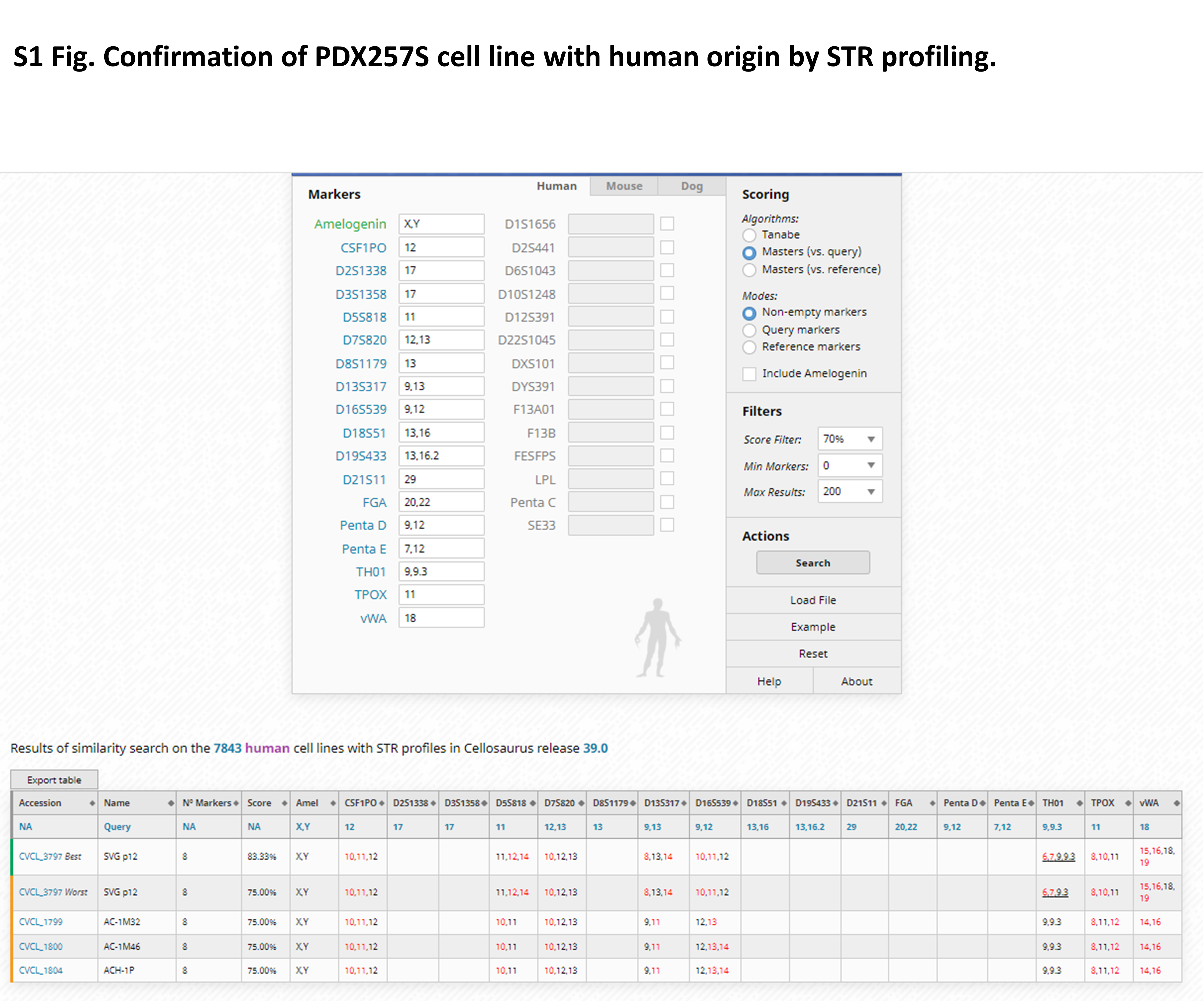

Supplement: Supplementary file 1 — Figure S1: Confirmation of PDX257S cell line with human origin by STR profiling. PDX257S cell line was submitted, and STR profiling was done at ATCC with confirmation report of human origin. STR profiles were also searched in the Cellosaurus database for potential matches to any human cell line (> 85%), as shown above. [file CAM4-14-e71150-s006.tif]

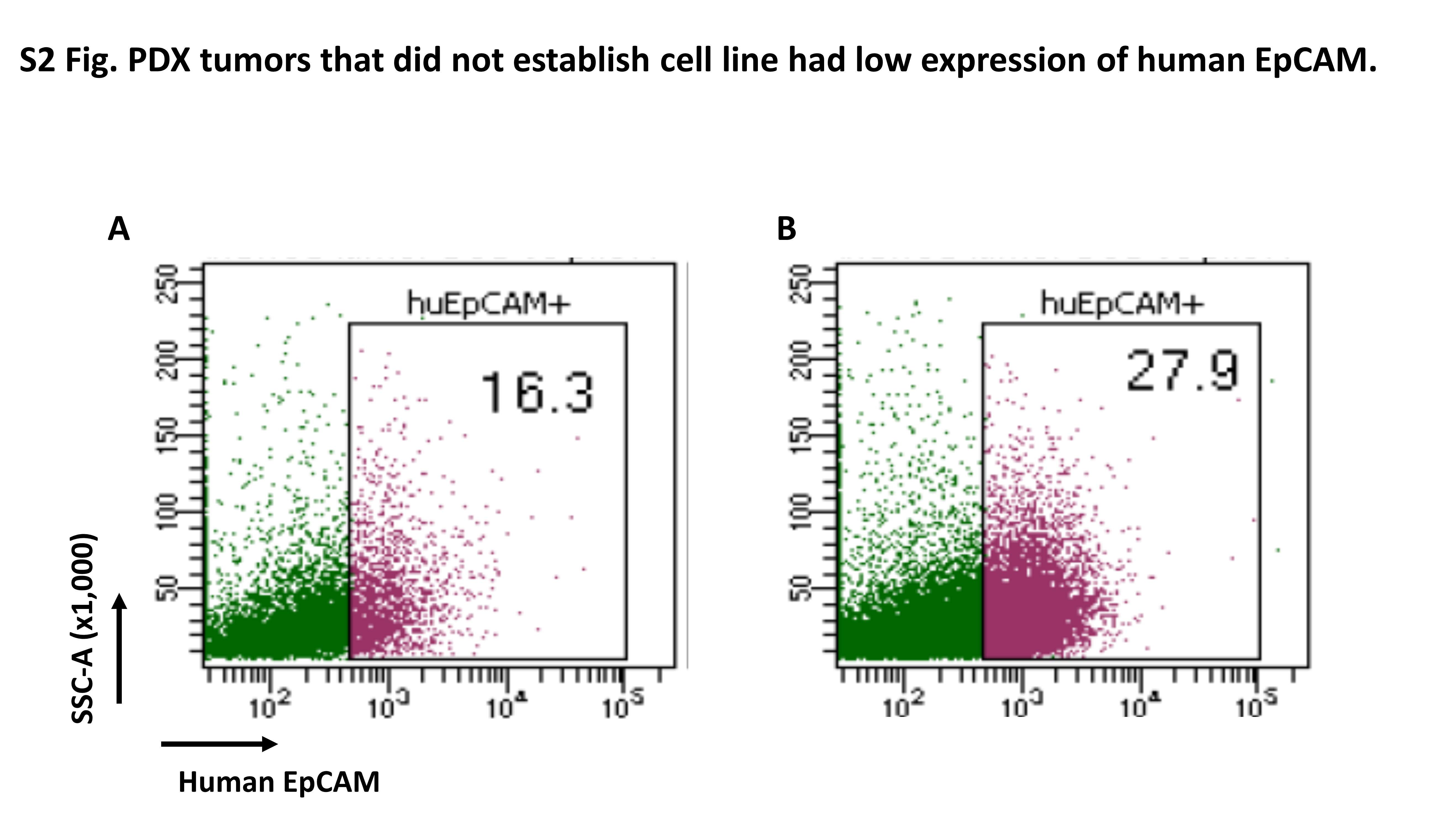

Supplement: Supplementary file 2 — Figure S2: PDX tumors that did not establish cell lines had low expression of human EpCAM. (A) Donor BCR347 and (B) donor BCR341 PDX early passage tumors (F1 or F2) had relatively low (< 50%) human EpCAM expression by flow cytometry analysis, whereas no subsequent cell lines were generated. [file CAM4-14-e71150-s003.tif]

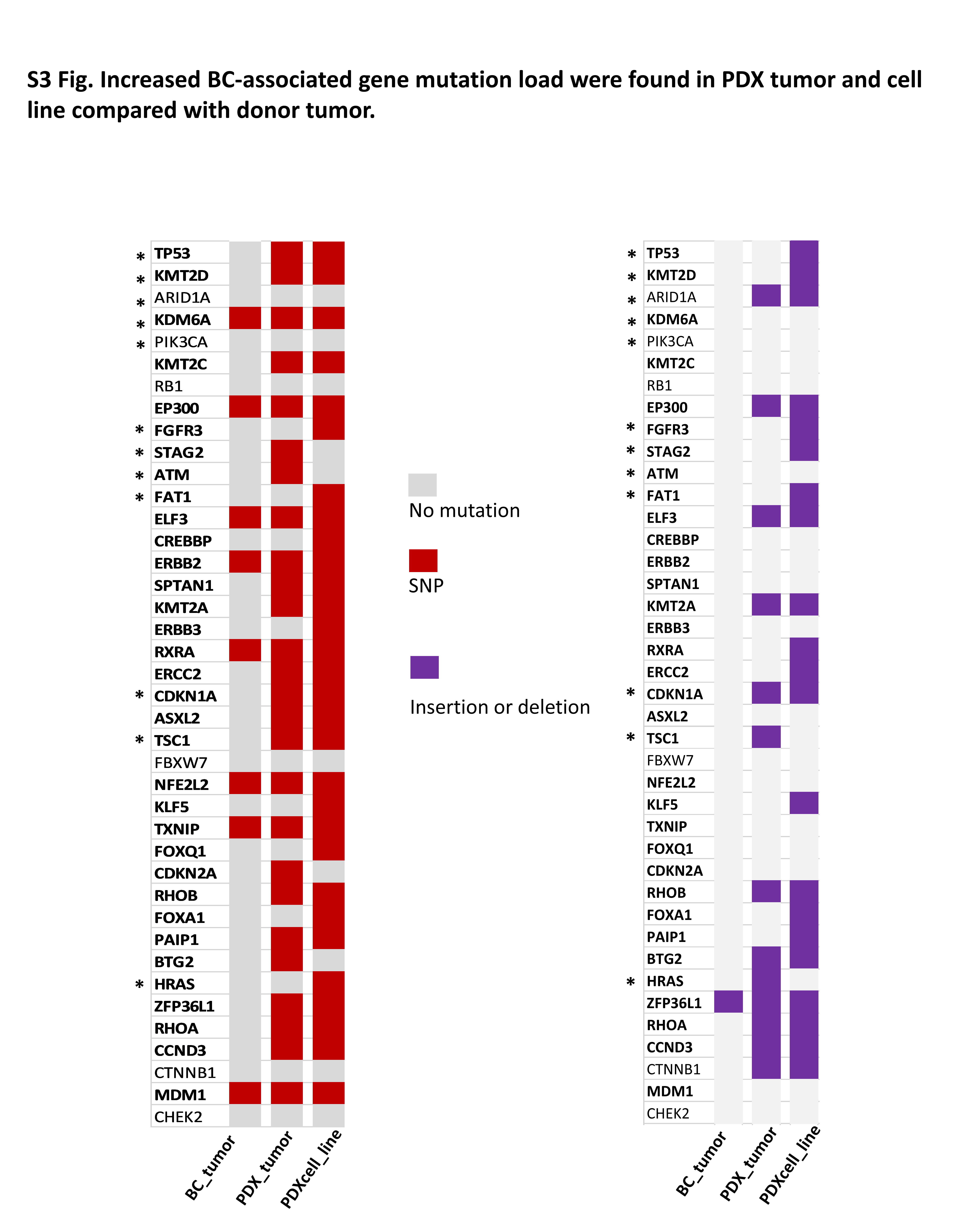

Supplement: Supplementary file 3 — Figure S3: Increased BC‐associated gene mutation load was found in the PDX tumor and cell line compared with the original tumor from the donor. Gene mutation analysis was performed for small nucleotide polymorphism (SNP; left panel, red blocks) and insertion/deletion type of mutations (right panel, purple blocks) for PDX tumor, cell line, as well as original donor tumor tissue. Gene names marked with asterisk represent oncogenic drivers or putative/potential drivers for BC. [file CAM4-14-e71150-s002.tif]

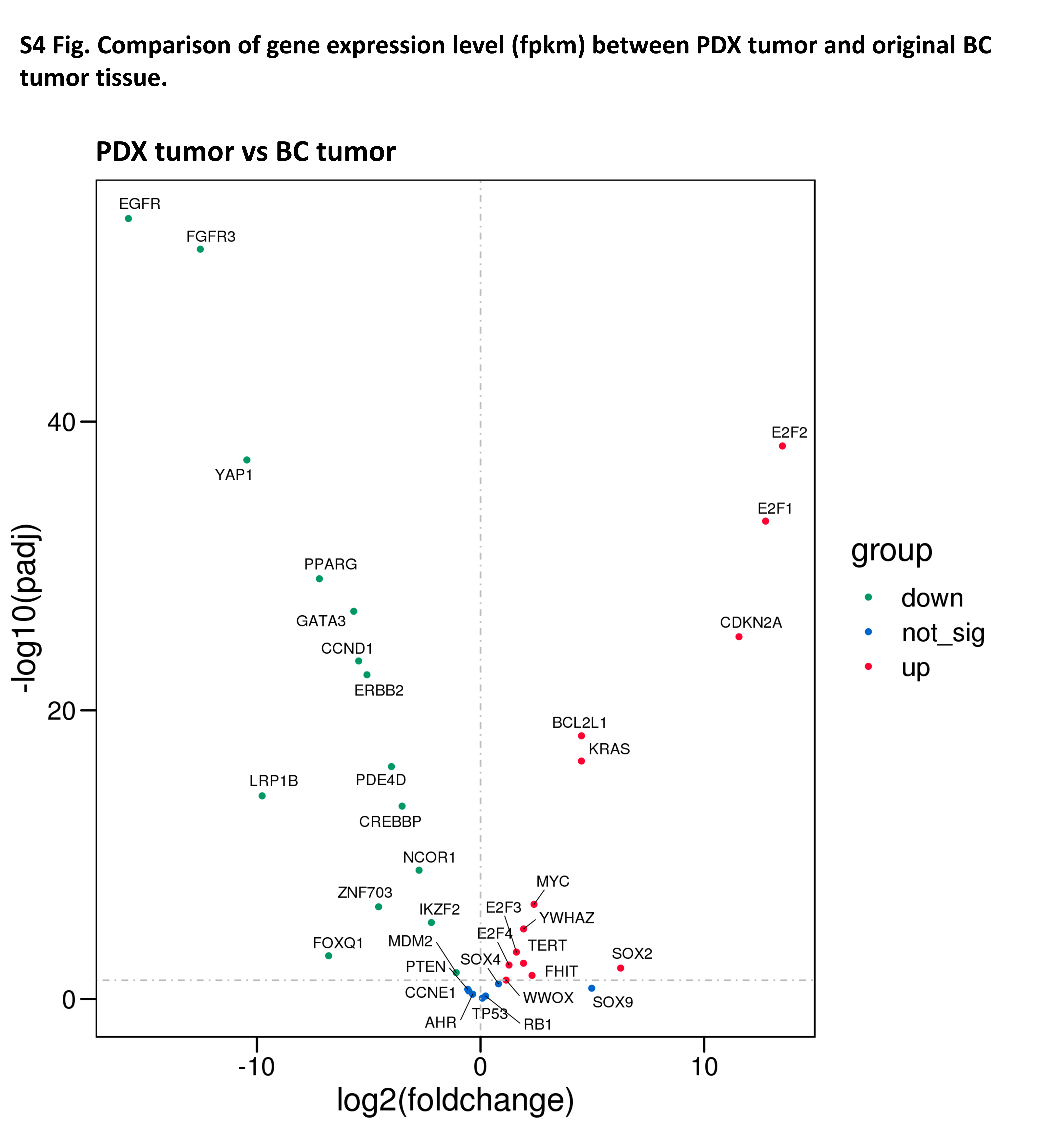

Supplement: Supplementary file 4 — Figure S4: Comparison of gene expression level (fpkm) between PDX tumor and original BC tumor tissue. Shown is a volcano plot indicating changes in the expression of those genes with relevance found in BC from published TCGA studies (either gene copy number or expressional changes). [file CAM4-14-e71150-s001.tif]

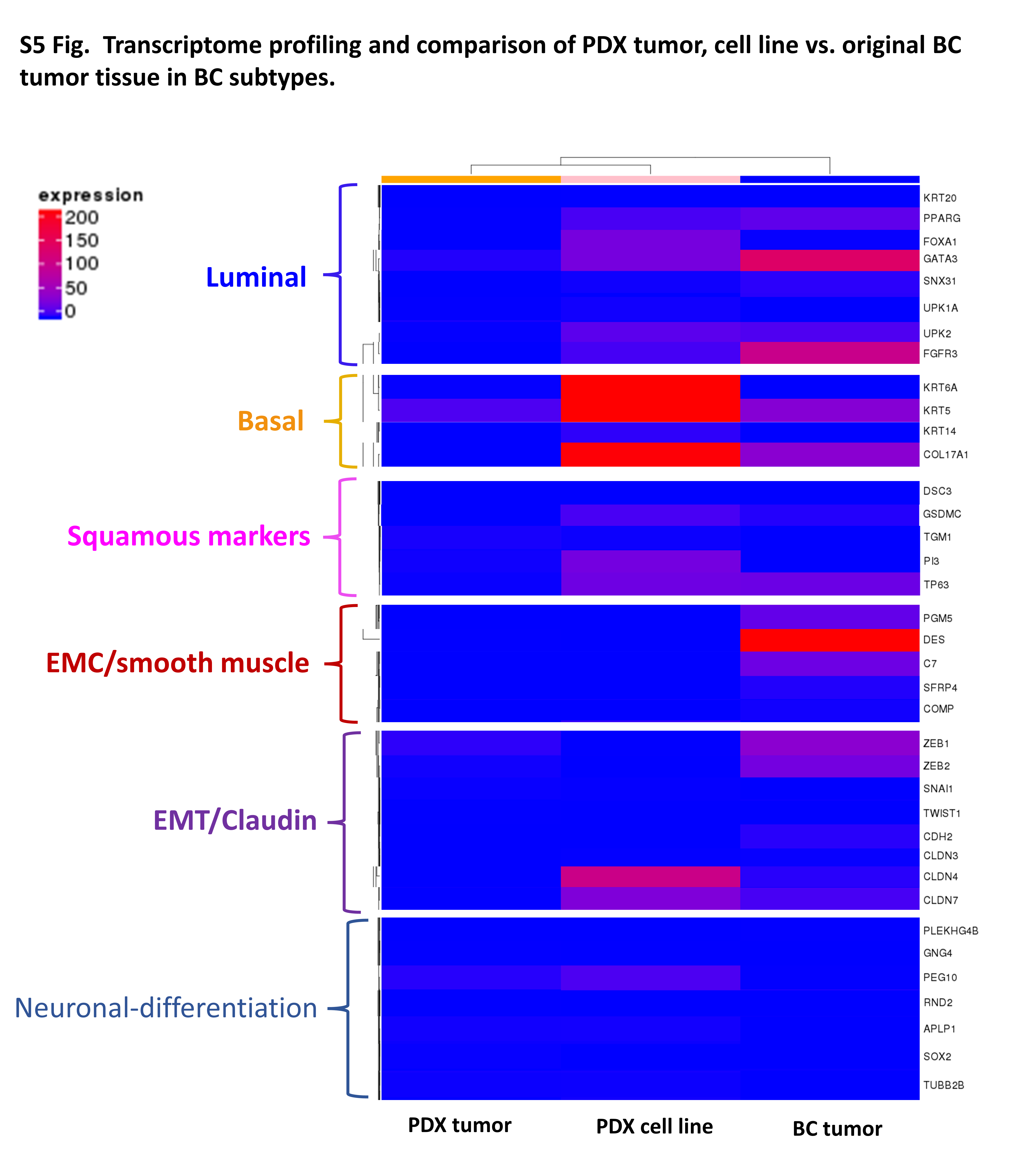

Supplement: Supplementary file 5 — Figure S5: Transcriptome profiling and comparison of PDX tumor, cell line versus original BC tumor tissue in BC subtypes. Shown is an expressional heatmap plotted on fpkm value, with clusters of genes indicating different subtypes of BC. [file CAM4-14-e71150-s007.tif]

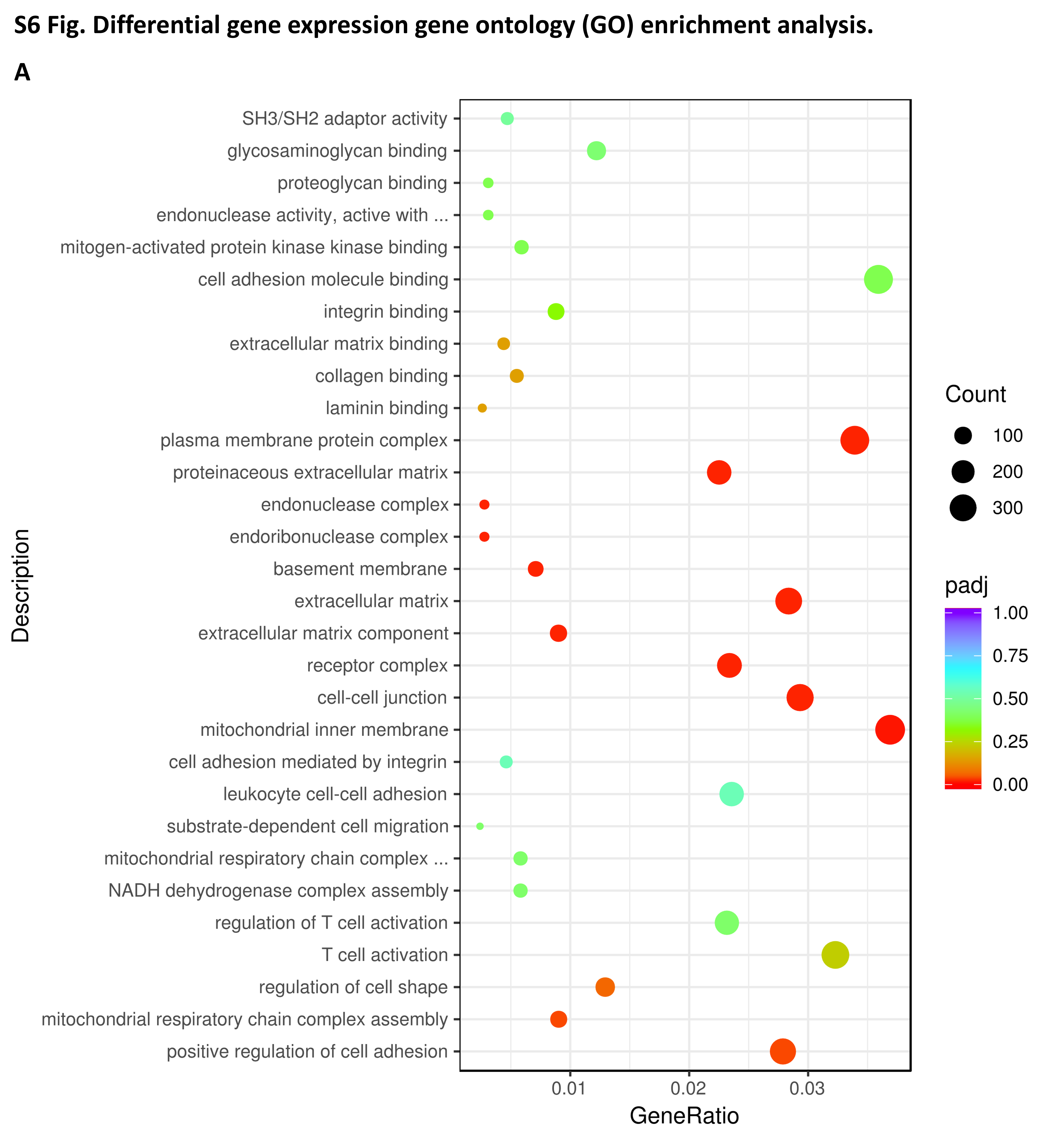

Supplement: Supplementary file 6 — Figure S6: Differential gene expression gene ontology (GO) enriched analysis of PDX tumor, cell line versus original BC tumor. Shown are GO enrichment analysis scatter plots for (A) PDX tumor vs. BC tumor and (D) PDX257S cell line vs. BC tumor. The abscissa in the graph is the ratio of the differential gene number to the total number of differential genes on the GO Term, and the ordinate is GO Term. padj: adjusted p‐value. Directed acyclic graphs (DAG) are also shown for (B‐C) PDX tumor vs. BC tumor or (E‐F) PDX cell line vs. BC tumor using GO terms under (B & E) cellular component and (C & F) molecular function. Each node represents a GO term, and the box represents the enrichment level of TOP5 GO Terms. The depth of the color represents the degree of enrichment; the darker the color is, the higher the enrichment degree is. Each node shows the name of the term and the padj of enrichment analysis. [file CAM4-14-e71150-s004.zip › cam471150-sup-0007-FigureS6@S6 FigA.tif]

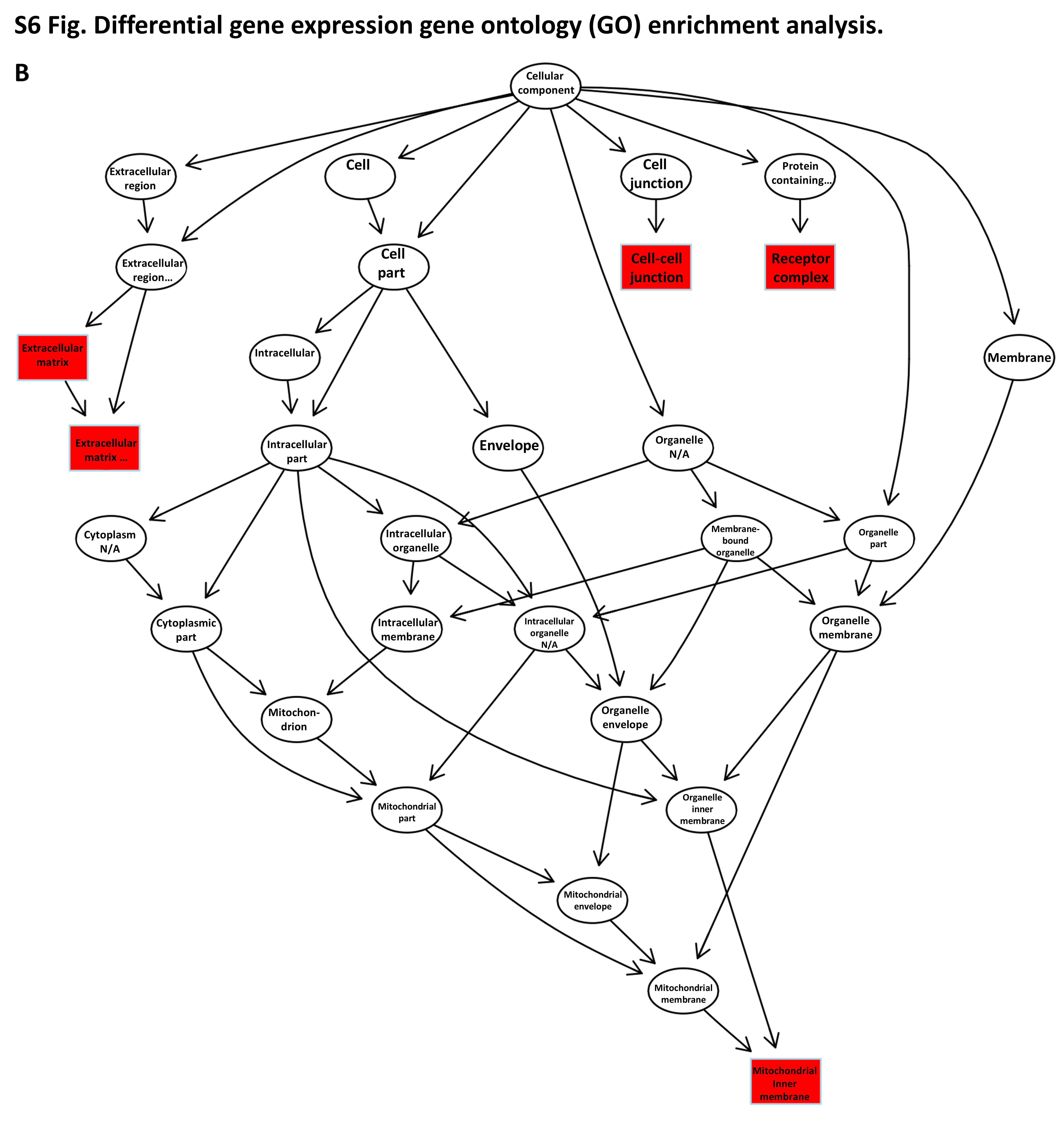

Supplement: Supplementary file 6 — Figure S6: Differential gene expression gene ontology (GO) enriched analysis of PDX tumor, cell line versus original BC tumor. Shown are GO enrichment analysis scatter plots for (A) PDX tumor vs. BC tumor and (D) PDX257S cell line vs. BC tumor. The abscissa in the graph is the ratio of the differential gene number to the total number of differential genes on the GO Term, and the ordinate is GO Term. padj: adjusted p‐value. Directed acyclic graphs (DAG) are also shown for (B‐C) PDX tumor vs. BC tumor or (E‐F) PDX cell line vs. BC tumor using GO terms under (B & E) cellular component and (C & F) molecular function. Each node represents a GO term, and the box represents the enrichment level of TOP5 GO Terms. The depth of the color represents the degree of enrichment; the darker the color is, the higher the enrichment degree is. Each node shows the name of the term and the padj of enrichment analysis. [file CAM4-14-e71150-s004.zip › cam471150-sup-0008-FigureS6@S6 FigB.tif]

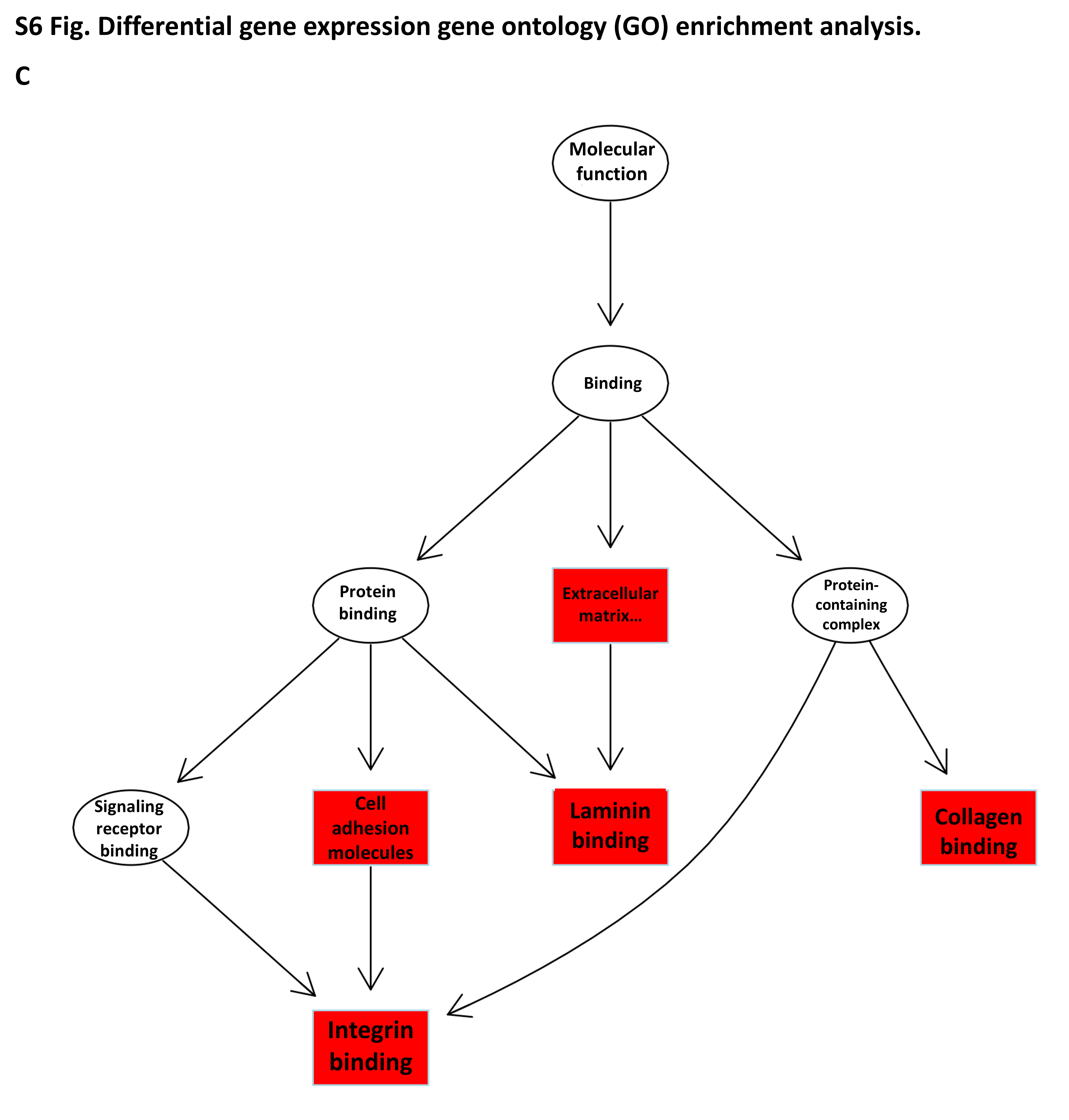

Supplement: Supplementary file 6 — Figure S6: Differential gene expression gene ontology (GO) enriched analysis of PDX tumor, cell line versus original BC tumor. Shown are GO enrichment analysis scatter plots for (A) PDX tumor vs. BC tumor and (D) PDX257S cell line vs. BC tumor. The abscissa in the graph is the ratio of the differential gene number to the total number of differential genes on the GO Term, and the ordinate is GO Term. padj: adjusted p‐value. Directed acyclic graphs (DAG) are also shown for (B‐C) PDX tumor vs. BC tumor or (E‐F) PDX cell line vs. BC tumor using GO terms under (B & E) cellular component and (C & F) molecular function. Each node represents a GO term, and the box represents the enrichment level of TOP5 GO Terms. The depth of the color represents the degree of enrichment; the darker the color is, the higher the enrichment degree is. Each node shows the name of the term and the padj of enrichment analysis. [file CAM4-14-e71150-s004.zip › cam471150-sup-0009-FigureS6@S6 FigC.tif]

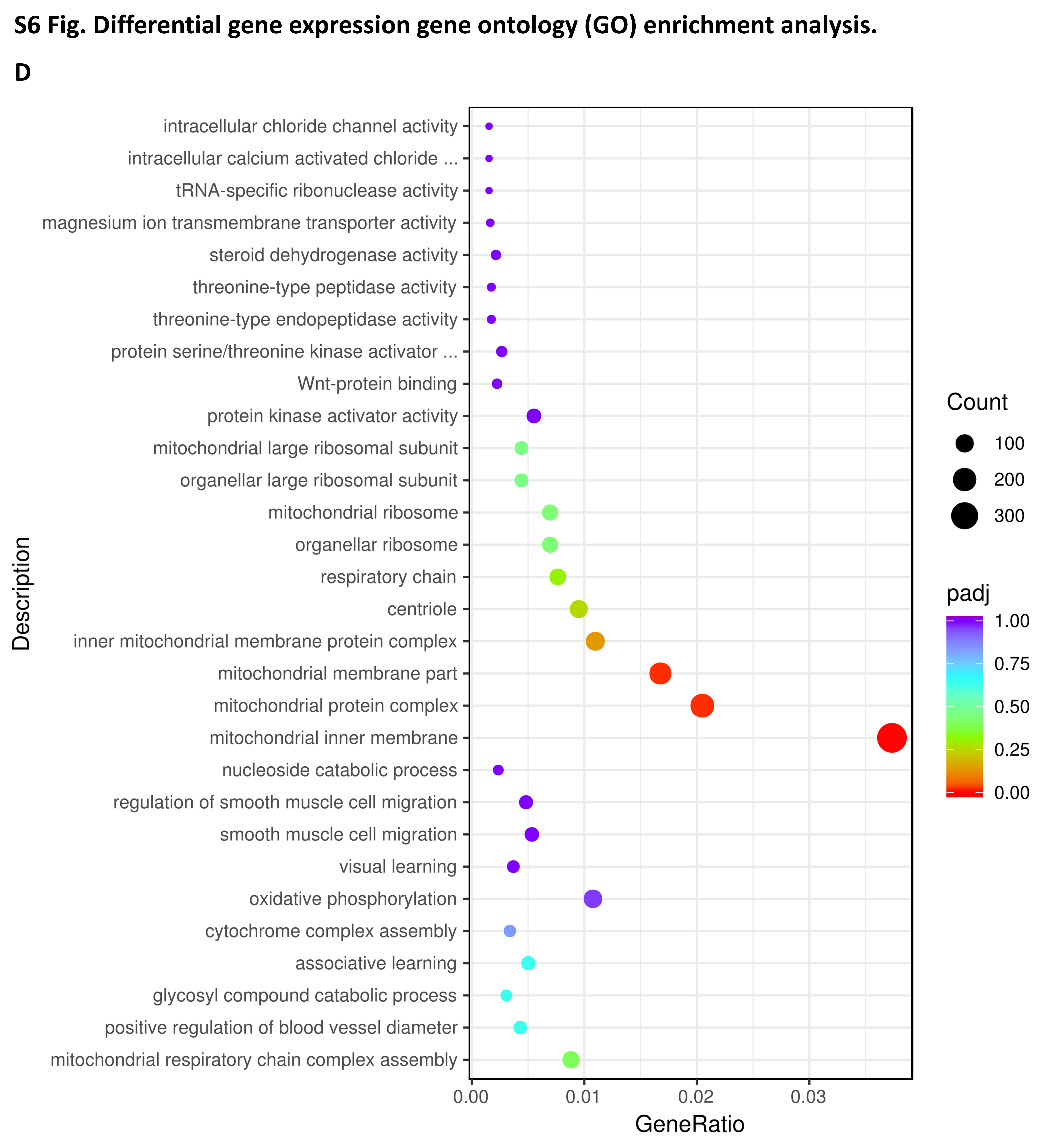

Supplement: Supplementary file 6 — Figure S6: Differential gene expression gene ontology (GO) enriched analysis of PDX tumor, cell line versus original BC tumor. Shown are GO enrichment analysis scatter plots for (A) PDX tumor vs. BC tumor and (D) PDX257S cell line vs. BC tumor. The abscissa in the graph is the ratio of the differential gene number to the total number of differential genes on the GO Term, and the ordinate is GO Term. padj: adjusted p‐value. Directed acyclic graphs (DAG) are also shown for (B‐C) PDX tumor vs. BC tumor or (E‐F) PDX cell line vs. BC tumor using GO terms under (B & E) cellular component and (C & F) molecular function. Each node represents a GO term, and the box represents the enrichment level of TOP5 GO Terms. The depth of the color represents the degree of enrichment; the darker the color is, the higher the enrichment degree is. Each node shows the name of the term and the padj of enrichment analysis. [file CAM4-14-e71150-s004.zip › cam471150-sup-0010-FigureS6@S6 FigD.tif]

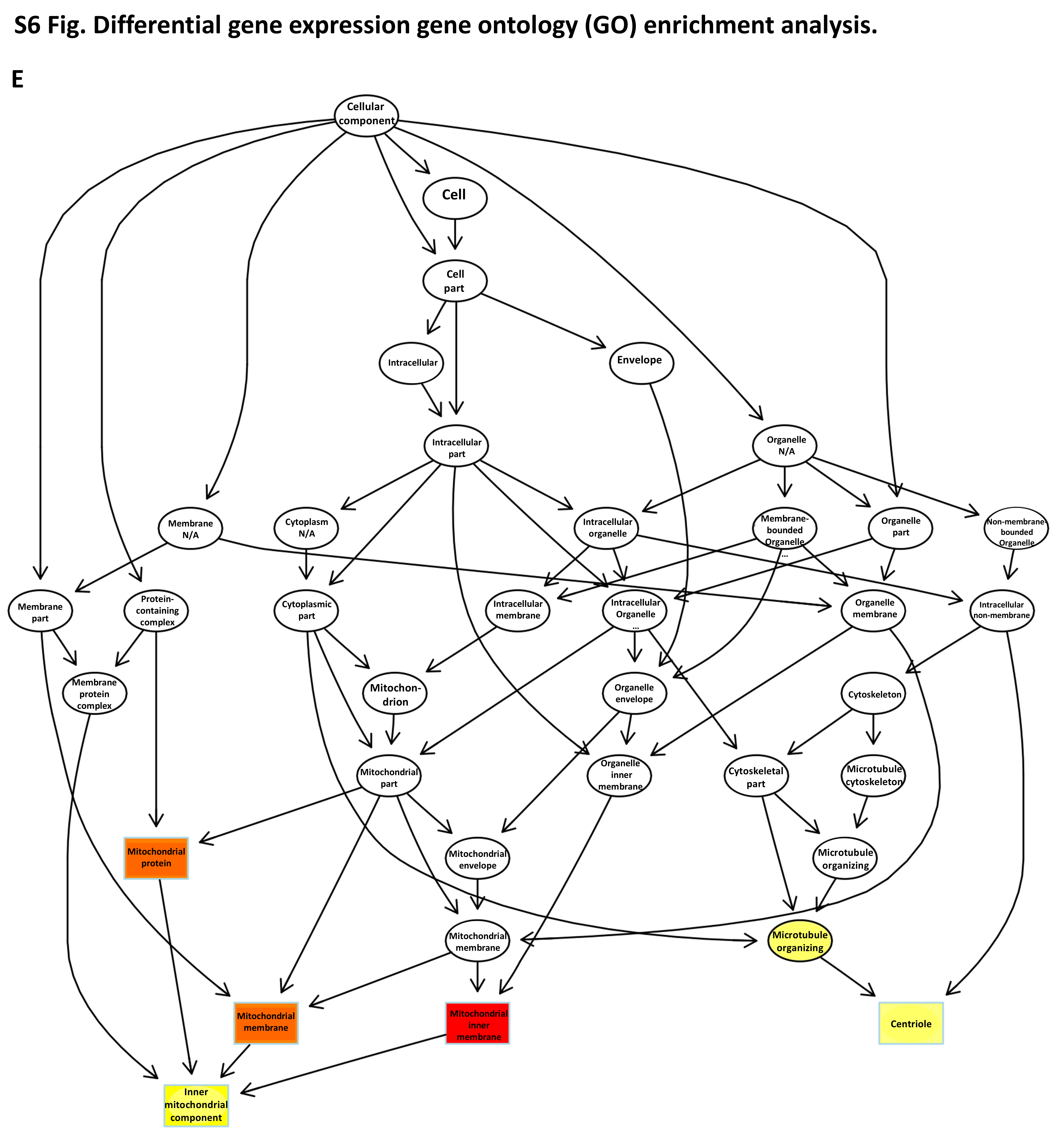

Supplement: Supplementary file 6 — Figure S6: Differential gene expression gene ontology (GO) enriched analysis of PDX tumor, cell line versus original BC tumor. Shown are GO enrichment analysis scatter plots for (A) PDX tumor vs. BC tumor and (D) PDX257S cell line vs. BC tumor. The abscissa in the graph is the ratio of the differential gene number to the total number of differential genes on the GO Term, and the ordinate is GO Term. padj: adjusted p‐value. Directed acyclic graphs (DAG) are also shown for (B‐C) PDX tumor vs. BC tumor or (E‐F) PDX cell line vs. BC tumor using GO terms under (B & E) cellular component and (C & F) molecular function. Each node represents a GO term, and the box represents the enrichment level of TOP5 GO Terms. The depth of the color represents the degree of enrichment; the darker the color is, the higher the enrichment degree is. Each node shows the name of the term and the padj of enrichment analysis. [file CAM4-14-e71150-s004.zip › cam471150-sup-0011-FigureS6@S6 FigE.tif]

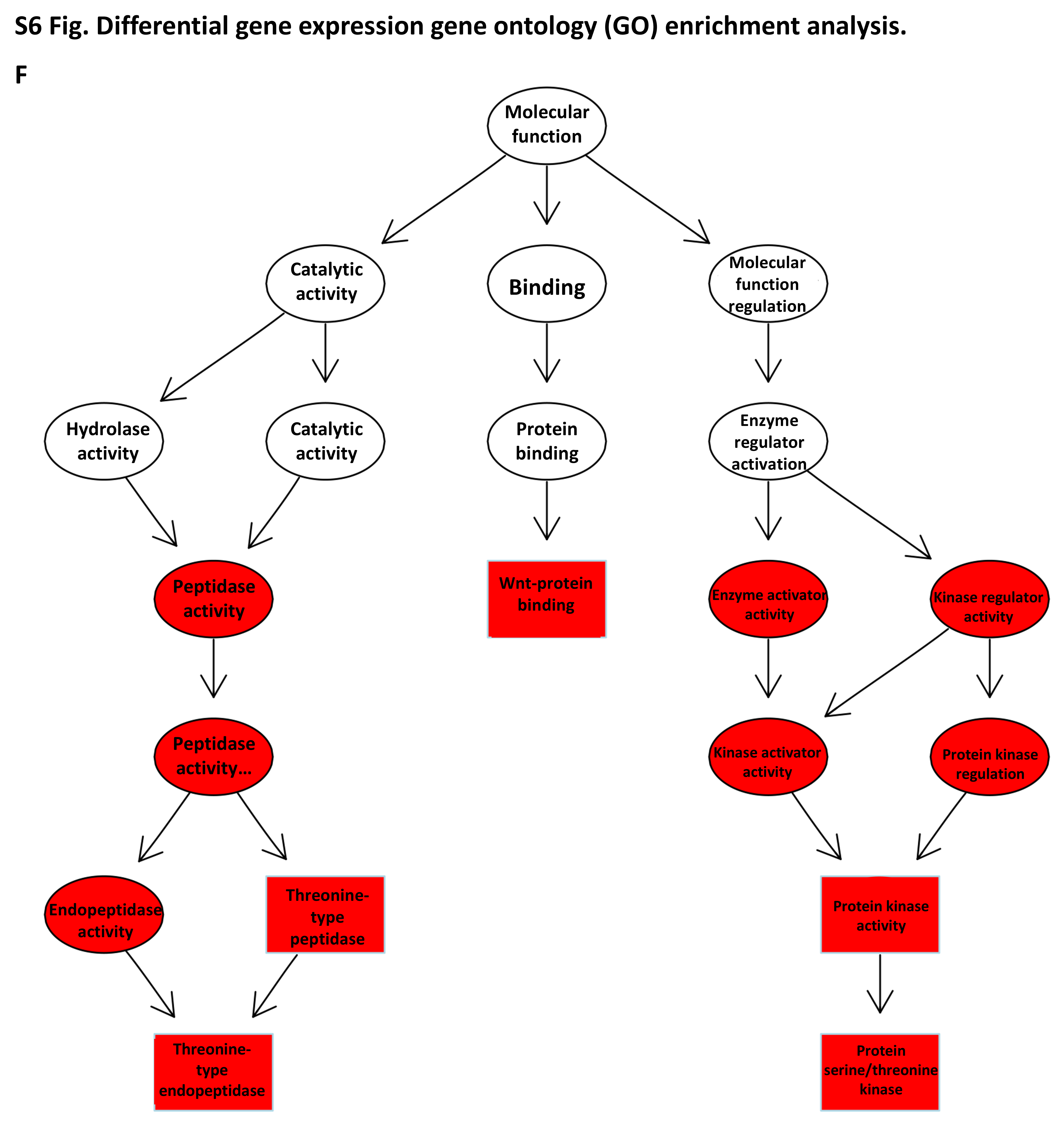

Supplement: Supplementary file 6 — Figure S6: Differential gene expression gene ontology (GO) enriched analysis of PDX tumor, cell line versus original BC tumor. Shown are GO enrichment analysis scatter plots for (A) PDX tumor vs. BC tumor and (D) PDX257S cell line vs. BC tumor. The abscissa in the graph is the ratio of the differential gene number to the total number of differential genes on the GO Term, and the ordinate is GO Term. padj: adjusted p‐value. Directed acyclic graphs (DAG) are also shown for (B‐C) PDX tumor vs. BC tumor or (E‐F) PDX cell line vs. BC tumor using GO terms under (B & E) cellular component and (C & F) molecular function. Each node represents a GO term, and the box represents the enrichment level of TOP5 GO Terms. The depth of the color represents the degree of enrichment; the darker the color is, the higher the enrichment degree is. Each node shows the name of the term and the padj of enrichment analysis. [file CAM4-14-e71150-s004.zip › cam471150-sup-0012-FigureS6@S6 FigF.tif]

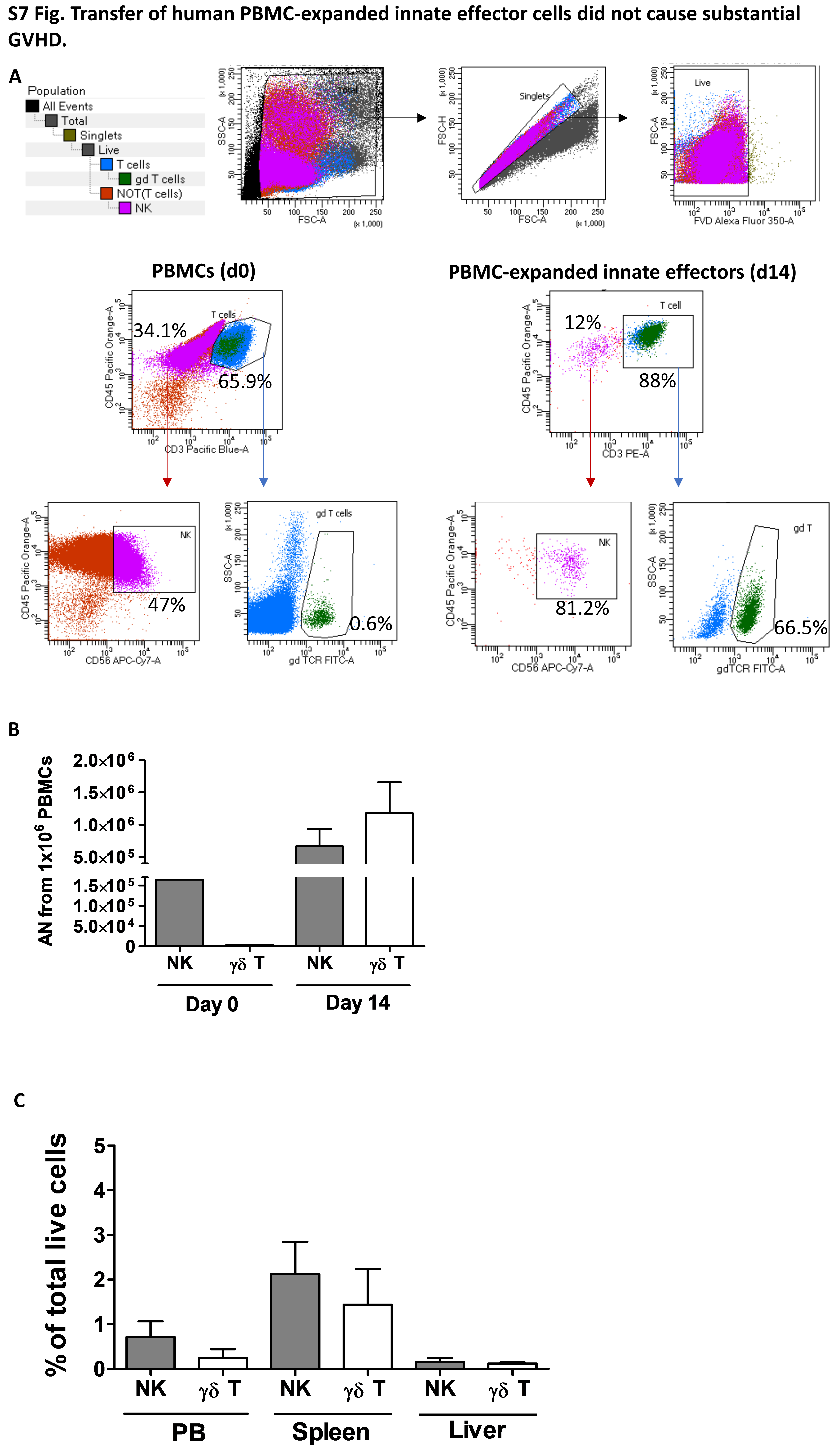

Supplement: Supplementary file 7 — Figure S7: Transfer of human PBMC‐expanded innate effector cells did not cause substantial GVHD. (A) Examples of flow cytometry gating strategy with (B) absolute number (AN) of innate effector cells (γδT cells and NK cells) per 106 PBMCs before and after expansion in vitro for 14 days. (C) Innate effector cells were injected subcutaneously together with PDX257S cells (ratio 2:1) into NSG mice for an in vivo cytotoxicity assay. By Day 40 post‐human innate effector cell engraftment, peripheral blood (PB), spleen, and liver were collected for flow cytometry analysis to detect human donor cells besides pathology staining. [file CAM4-14-e71150-s008.tif]
