# Supplementary material for: A Double‐Humanized Murine Model in Bladder Cancer: A Novel Preclinical Model for Cancer Immunology Research
Source: Cancer Med. 2025 Aug 13;14(15):e71150. doi: 10.1002/cam4.71150 (PMC12344512; doi:10.1002/cam4.71150)
Supplement: Supplementary file 8 — Table S1: Donor tumors with successful PDX implants were mostly from invasive and/or high‐grade urothelial carcinoma. [file CAM4-14-e71150-s005.docx]

**S1 Table. Donor tumors with successful PDX implant were mostly from invasive and/or high-grade urothelial carcinoma.**
